# Synergy between trastuzumab and pertuzumab for human epidermal growth factor 2 (Her2) from colocalization: an *in silico *based mechanism

**DOI:** 10.1186/bcr2888

**Published:** 2011-05-22

**Authors:** Gloria Fuentes, Maurizio Scaltriti, José Baselga, Chandra S Verma

**Affiliations:** 1 Bioinformatics Institute (A*STAR), 30 Biopolis Street, #07-01 Matrix, 38671, Singapore; 2 Division of Hematology and Oncology, Massachusetts General Hospital Cancer Center, Harvard Medical School, 10 North Grove Street, LH-108, Boston, MA 02114, USA; 3 Medical Oncology Department, Vall d'Hebron Institute of Oncology (VHIO), Vall d'Hebron University Hospital, Passeig Vall d'Hebron 119-129, Barcelona 08035, Spain; 4 Department of Biological Sciences, National University of Singapore, 14 Science Drive 4, 117543, Singapore; 5 School of Biological Sciences, Nanyang Technological University, 60 Nanyang Drive, 637551, Singapore

## Abstract

**Introduction:**

Human epidermal growth factor 2 (Her2), a receptor tyrosine kinase, is overexpressed in breast cancers. It has been successfully targeted by small molecule kinase inhibitors and by antibodies. Recent clinical data show a synergistic response in patients when two antibodies, trastuzumab and pertuzumab, are given in combination.

**Methods:**

This unexpected effect is rationalized through computer models and molecular dynamic simulations by hypothesizing that the two antibodies can co-localize on the same molecule of the Her2 extracellular domain.

**Results:**

Simulations suggest that the clinical synergism observed for the two antibodies arises partly from enhanced affinity that originates in cooperative interactions between these two antibodies when they are co-localized on Her2 and "clamp" it; this may inhibit dimerization and possibly higher oligomerizations with neighboring receptors. In the presence of trastuzumab, the receptor becomes highly plastic, especially domains I to III, and this appears to promote increased association with pertuzumab. Further, the presence of pertuzumab evokes novel interactions between the receptor and trastuzumab. Indeed, splicing out of this region *in silico *results in a big reduction in the interactions of the antibody with the receptor.

**Conclusions:**

If validated, these findings will bring about a new direction in the design of antibodies whereby different epitopes on the same antibody may be targeted to lead to synergistic/cooperative inhibition and contribute to generate more potent therapeutics and to increase clinical efficacy.

## Introduction

**Figure 1:**
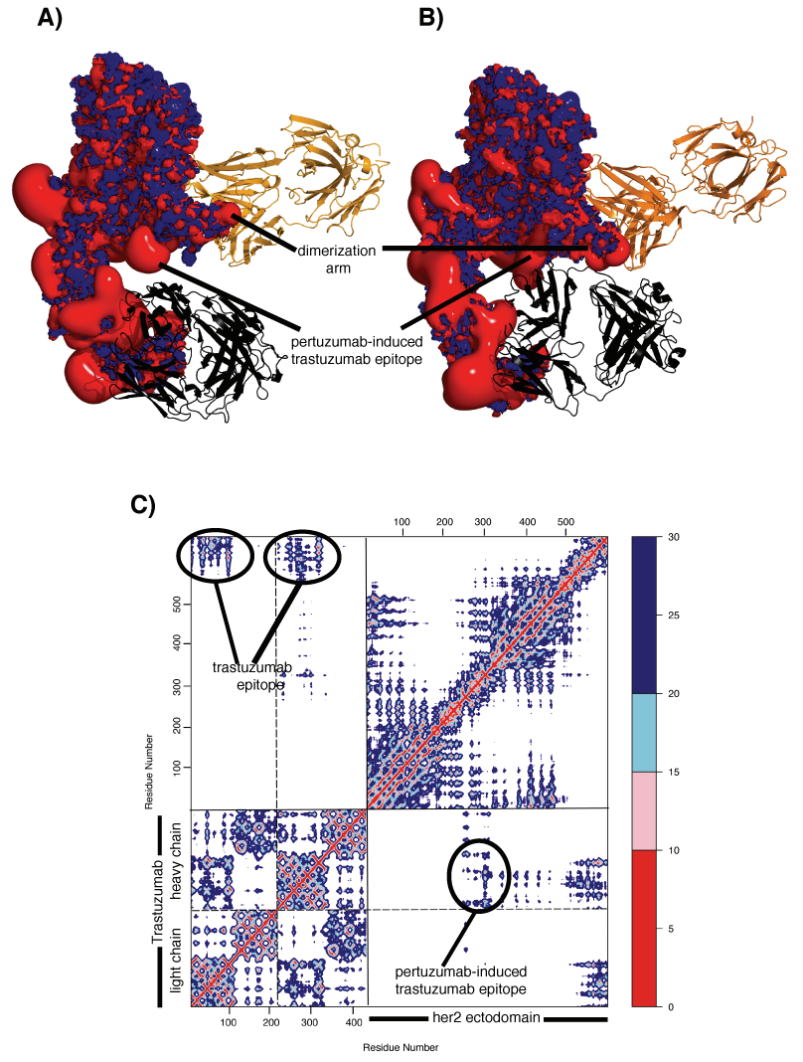
**Representation of the time evolution of the Her2 and Abs complex and their interactions**. Representation of the *in silico *pertuzumab (in orange) -induced trastuzumab (in black) epitope that emerges during the course of the MD simulations of Her2 ectodomain (in red-blue electrostatic surface where red represents regions of acidity and blue represents regions of basicity) in presence of trastuzumab and/or pertuzumab. **(A) **Her2:trastuzumab complex structure at the beginning of the simulation(time = 0 ns) showing no contact between trastuzumab and the new epitope and **(B) **snapshot taken during the MD simulation (time = 15 ns) showing interactions between trastuzumab and the new epitope; **(C) **dynamic Cα-Cα contact matrix (representing the distance between each pair of residues comprising the three dimensional structure of protein complex), the data from both pertuzumab and trastuzumab co-localized on Her2 are shown below the diagonal; the data from the Her2-trastuzumab complex are shown above the diagonal. Trastuzumab has been coloured in black; while pertuzumab is shown in orange. This plot shows the occurrence of interactions between the heavy chain of trastuzumab (see vertical axis) and the region of the ectodomain around 310 to 330 (see the horizontal axis). During the course of simulation, when pertuzumab is bound to Her2:trastuzumab these contacts are formed, in contrast to the simulation where only Her2:trastuzumab are present (comparison with the same region above the diagonal) where these contacts are absent.

ErbB receptors are the prototypical founders of the growth factor receptor tyrosine kinase (RTK) family. They are activated by the binding of different ligands and are involved in the transmission of signals from the extracellular space to the cytoplasm and nucleus of a cell, thus orchestrating biological processes [1]. Among the members of this family, which also includes the epidermal growth factor receptor (EGFR or ErbB1), ErbB3, ErbB4, Her2 or ErbB2 is homologous to, but distinct from the others, since it is not activated upon ligand binding. This ligand-independent activation makes Her2 the universal heterodimeric partner for each of the other ErbB family members [2]. The architecture of these receptors [3,4] reveals an extracellular domain (made up of four subdomains), a single transmembrane helix, and an intracellular domain (consisting of a juxtamembrane region, a tyrosine kinase domain, and a C-terminal tail harboring autophosphorylation sites that serve as docking sites for adaptor proteins [5,6]). The importance of the tight regulation of these receptors and in particular Her2 is signified in human breast cancers, where Her2 is overexpressed by approximately 20 to 30% and this is normally associated with more aggressive tumours and a poorer prognosis [7,8]. Due to this active role in human cancers, a number of therapeutic approaches are currently under development to block the effects of Her2 overexpression, including kinase inhibitors (such as lapatinib) and monoclonal antibodies (trastuzumab and pertuzumab) [9]. The anti-Her2 monoclonal antibody trastuzumab binds to domain IV of Her2, a region that is not involved in receptor dimerization and is thought to work in a complex manner [10,11]. In contrast, pertuzumab binds to Her2 near the centre of the domain II dimerization arm (Figure 1). The overlap between the pertuzumab epitope and the probable heterodimer interface suggests steric occlusion by physically blocking the formation of Her2-containing heterodimers. Recent clinical observations have demonstrated that combining trastuzumab and pertuzumab together yields surprisingly synergistic results in tumour inhibition (that is, the combined effect of using the two molecules was far greater in effect than when using either alone) during the treatment of Her2 positive breast cancers that progressed during prior trastuzumab therapy [12]. This suggests a cooperative mechanism of inhibition that can lead to clinical improvement in the treatment of these tumours.

We have used molecular modelling to develop a mechanism that may partly explain this cooperative effect. A simple model is that cooperativity could arise from the physical co-localization of the two antibodies on the same Her2 molecule, as has been previously demonstrated for the insulin-like growth factor receptor [13]. Models of the binary structures of Her2:trastuzumab and Her2:pertuzumab were combined to create a model of the ternary complex of Her2 and the two Fab fragments of these antibodies. This system was subjected to structural/energetic refinement followed by molecular dynamics (MD) simulations (Additional file 1).

## Methods

### Building the models

The binary associations of Her2 and the two antibodies have been experimentally characterized and are available in the Protein Data Base (pdb entries: 1n8z[3] and 1s78 [14]). The electronic density of the C-terminal region of the receptor is the Her2:pertuzumab complex is missing, due probably to the high inherent flexibility of this part of the structure. For the coordinates of the residues in this region, we have assumed that this stretch will adopt a conformation similar to the one found in the complex with trastuzumab and have been so modeled. A few other missing residues, located in loops, have been modeled based on homologue structures. The ternary model of the complex Her2 and the two Fab domains of these antibodies, trastuzumab and pertuzumab, were manually constructed using the coordinates of the previous models, after superposition on the backbone of the receptor ectodomain.

### MD simulations and binding energy calculations

We have used seven different systems for this study (Additional file 1); the complete 3D model structure of the complex (Her2-P-T), the receptor bound to trastuzumab (Her2-T) and pertuzumab (Her2-P), the apo-receptor (Her2), the two independent antibodies (pert and trast) and both antibodies co-localized as they are found in the complex (Abs). All these systems have been refined using several energy minimization and short MD simulation cycles in order to equilibrate the structures after which they were subjected to the production phases of MD simulation (around 18 to 20 ns each) in explicit water, as detailed. The AMBER (Assisted Model Building with Energy Refinement) 9 package [15] together with the ff99 force field [16] was used. The protonation states of the residues were assigned using PDB2PQR [17], after which the systems were surrounded with a box of TIP3 or Transferrable Interaction Potential 3 water molecules that result in an 8 Å layer of water molecules around the proteins and neutralized by the necessary numbers of Cl-or Na+ counter-ions using the t-leap module of AMBER 9. The solvated systems were then subjected to a minimization phase, mainly by the steepest descend method and small number of conjugate gradient steps, in order to remove bad contacts. After that a 50 ps heating phase was carried out, where the proteins were heated from 0 to 300 K, followed by another 50 ps phase density equilibration at a constant pressure of 1 atm. A final 2 ns unconstrained MD simulation was carried out. The production runs were extended to around 20 ns at constant temperature using a Langevin thermostat [18] and constant pressure (1 atm) by coupling to a Berendsen barostat These two queries do not need to be address since these are mathematical methods and not devices [19]. The particle mesh Ewald (PME) method [20] was used with a direct-space non-bonded cutoff of 8 Å to account for long range interactions. Covalent bond lengths to hydrogen atoms were constrained using the SHAKE algorithm [21], which allowed a time step of 2 fs for all the MD simulations. Snapshots in the interval of 8 to 16 ns were extracted for further processing. The analysis was carried out using functionalities in the ptraj module of AMBER 9, the module Bio3D [22] of R and Dyndom [23]. The PyMOL molecular viewer, a software package [24] was used for visualizing and generating the figures.

The binding free energy ΔG_bind _of the different systems was calculated according to the so-called Molecular Mechanics/Generalized-Born Surface Area (MM/GBSA) procedure [25]. Here ΔG_bind _denotes the contributions from the molecular mechanics (MM) energies of the molecules, and originates in contributions from internal energies, electrostatics (ΔG_ELE_) and van der Waals (ΔG_VDW_) contributions. These terms have been calculated using the mm_pbsa module of AMBER 9. The polar component was evaluated with the Generalized-Born (GB) approach. In this approach, the non-polar contribution to the solvation energy was calculated as ΔG_NP _= γ (SASA) + β, in which γ = 0.00542 kcal/Å, β = 0.92 kcal/mol and SASA is the solvent-accessible surface area estimated with LCPO (linear combination of pairwise overlaps) method [26] as implemented in AMBER. While the real free energy of a system also includes contributions from the entropies, given the large sizes of the systems studied here, computations of entropies were prohibitive and hence could not be carried out. However, the free energies computed here have been averaged over several conformations generated through MD simulations, and hence the effects of entropy to some extent are implicitly included in the current calculations.

### Essential dynamics analysis and Dyndom analysis of Her2 and its antibodies

Essential dynamics [27,28] have been widely used for filtering large scale concerted fluctuations from an ensemble of conformations, like those obtained from MD simulations [29]. The method is comparable to a principal component analysis, and it is based on the construction of the diagonalized covariance matrix containing the atomic fluctuations. We have performed a standard essential dynamics (or Principal Component Analysis, PCA) analysis in order to identify the most relevant motions occurring in the receptor in the different bound states considered on the MD simulations carried out in this study (Additional file 2); all the covariance matrices have been calculated taking the apo receptor as a reference structure. This yields a set of eigenvectors in a 3N-dimensional space, and eigenvalues, reflecting the magnitude of the fluctuations along the corresponding eigenvectors. Each eigenvector describing a collective motion can be represented by two structures, one at the maximum value, the other at the minimum value of the collective variable when the trajectory is projected onto it. These two structures thus generated for the first eigenvector of each trajectory were used as input for the program DynDom [23], using default parameters, in order to determine dynamic domains, hinge axes and hinge-bending regions.

## Results and discussion

### Conformational space of Her2 in different bound forms

Simulations show that the ectodomain of Her2 adopts various conformations, depending on its bound state. The binary states (when complexed to trastuzumab or pertuzumab) are more stable compared to the apo or the ternary complex (Additional file 3). This could stem from the high intrinsic flexibility of the apo receptor or/and the ternary complex. Although a complete understanding of the behaviour of such a receptor demands the full-length structure, we believe that the models used here represent valid starting structures for the type of analyses carried out in this study. As expected, the presence of an antibody immobilizes its corresponding epitope (Additional file 4). The largest fluctuations in the pertuzumab binding region (ranging from 240 to 300) and in the trastuzumab epitope (around 550 to 600) are seen in the apo state or in the Her2:trastuzumab or Her2:pertuzumab complexes respectively. Interestingly the dimerization arm, a β hairpin in domain II that protrudes from the rest of Her2, together with the spatially adjacent regions, undergoes enhanced fluctuations when trastuzumab binds. This may reinforce the fact that the Her2:trastuzumab complex can actually promote the association of Her2 with other members of the ErbB family.

We have also investigated the flexibility of the antibodies and the stability of their binding interfaces with Her2. In general, the complementary determining regions (CDR) exhibit smaller fluctuations in comparison to the rest of the antibody; whereas regions far from the receptor's influence remain extremely flexible. In the ternary complex, the C-terminal half of the light chain of pertuzumab shows an increased flexibility, induced by the presence of trastuzumab (Additional file 4). Trastuzumab shows a more complicated pattern. When only trastuzumab is present in the complex, the whole antibody shows a high flexibility, with the exception of the CDR for the light chain. In the ternary complex, the light chain undergoes the largest fluctuations in the region interacting with the receptor binding epitope; however the most relevant difference is an attenuation in the mobility of the N-terminal half of the heavy chain due to the proximity of one of the disulphide-bonded modules of domain II, which in turn shows lower flexibility (residues 310 to 325; see section below on new epitopes). We can conclude that the dynamics of pertuzumab seem to be regulated mainly by the binding to the receptor; while for trastuzumab, the co-binding of the second antibody has a significant effect. When the two antibodies are analysed in the absence of the receptor, trastuzumab *per se *seems more flexible than pertuzumab, which can be the origin of the more complex dynamics observed for the systems including this antibody. These intricate changes and regulation between the different components of the system reflect the importance of flexibility and could point to "allosteric" changes in explaining the *in vivo *synergistic effect.

### Uncovering new epitopes in Her2

The analysis of the interactions between Her2 and the two antibodies reveals that the perturbation of the crystallographically observed interfacial region of Her2 is small during the simulations of the binary complexes (Additional file 5). However, the co-localization of the two Abs on Her2 surprisingly triggers the formation of additional and novel contacts of Her2 with trastuzumab; these lie outside of its epitopes (Figure 1). This β-hairpin loop region, termed the pertuzumab-induced trastuzumab epitope, is located in domain II and is in proximity to the dimerization arm. The requirement of the co-localization of the two Abs for the formation of this new epitope is revealed in the minimum distance matrices for all the Cα atoms for the receptor and trastuzumab in the simulations of the binary and ternary complexes (see Figure 1C upper and lower diagonal of the matrix, respectively). This can also be observed from the reported distances between two residues in this novel epitope and residues at the interface in trastuzumab (Additional file 6). For this epitope to emerge in the Her2:trastuzumab interface, the presence of pertuzumab is strictly required. This novel association is mainly driven by long-range interactions, and could be the origin of the observed synergism.

### Quantification of synergism between pertuzumab and trastuzumab

The MM-GBSA (molecular mechanics Generalized Born surface area) [25] is regularly employed to obtain estimates of the free binding energy of large systems and these are largely reasonable [30-32]. These only refer to the enthalpic component of the overall free energies of binding because computations of entropies of such large systems are prohibitively expensive. The derived association energetics of trastuzumab and pertuzumab to Her2 are shown in Table 1; the computed values are very large as is the case with computations involving large systems; indeed in comparison the binding of trastuzumab to the extracellular domain of Her2 and to Her2 positive cells ranges from approximately -12.5 to -14 kcal/mol [33]. However, despite being large, qualitative analyses of the differences in the computed binding energies are very useful in ranking interactions. Our findings suggest (the more negative an energy, the more stable the associated system):

**Table 1:** *In silico *binding free energy for the association of Her2 ectodomain and the two antibodies.

	ΔG_ELE_	ΔG_VDW_	ΔG_GB_	ΔG_NP_	Total ΔG
Her2-T < -Her2 + trast	-841.6	-116.3	-197.8	-14.8	-1,144.6
Her2-P < -Her2 + pert	-490.5	-55	-480.1	-4.3	-1,095.7
Her2-P-T < -Her2 + trast + pert	-1,028.8	-102.6	-217.7	-11.5	-1,438.8
Her2-P-T < -Her2 + Abs	-1,374.4	-114.9	730.4	-17.6	-851.0
Her2-trunc-P-T < -Her2-trunc + trast + pert	-1,044.3	-149.4	469.2	-20	-725.0

Binding free energy components (kcal/mol) for the association of Her2 to pertuzumab and trastuzumab calculated using MM-GBSA method using MD snapshots extracted from independent simulations.

Her2-T, Her2-trastuzumab; trast: trastuzumab; Her2-P, Her2-pertuzumab; pert, pertuzumab; Her2-P-T, Her2-pertuzumab-trastuzumab; Abs, antibodies (here specifically it refers to both pertuzumab and trastuzumab); Her2-trunc, truncated form of Her2; ΔG_bind_, binding free energy; MM/GBSA, molecular mechanics/Generalized-Born surface Area; ΔG_ELE_, electrostatics energy; ΔG_VDW_, van der Waals energy; GB, Generalized-Born; ΔG_NP_, non-polar energy

**Figure 2:**
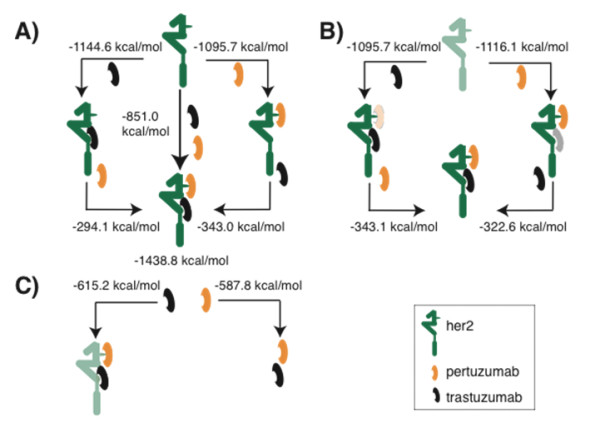
**Thermodynamic cycles to study the cooperative binding of Her2 to trastuzumab and pertuzumab**. **(A) **The cycle with the energy estimations calculated from the independent association simulations. **(B) **The cycle analysed using the conformations of receptor and binary complexes from the co-localized interaction simulations. The energetics for the binary complexes have been obtained from the ternary complex of Her2:pertuzumab:trastuzumab complex, in an attempt to account for the influence of the other antibody when both are co-localized. **(C) **The cycle representing the gain in energy between the binding of the two antibodies to each other in an isolated system or when co-localized onto the structure of the receptor. The ectodomain of Her2 has been coloured in green, while trastuzumab and pertuzumab are represented in black and orange, respectively. The differences in free energy binding estimated for every system are shown. The computations were for the following schemes: A) her2-trastuzumab < -her2+ trastuzumab; her2-trast-pert < -her2-trast + pertuzumab her2-trast + pertuzumab < -her2+ trastuzumab + pertuzumab her2-pertuzumab < -her2+ pertuzumab; her2-trast-pert < -her2-pert + trastuzumab. B) her2-trastuzumab (extracted from her2:pert:trast) < -her2+ trastuzumab; her2-trast-pert < -her2-trast (extracted from her2-pert:trast) + pertuzumab her2-pertuzumab (extracted from her2:pert:trast) < -her2+ pertuzumab; her2-trast-pert < -her2-pert (extracted from her2-pert:trast) + trastuzumab. C) Abs (extracted from her2:pert:trast) < -pertuzumab + trastuzumab; Abs < -pertuzumab + trastuzumab.

- trastuzumab has a higher affinity for apo Her2 than pertuzumab (Figure 2A); this arises from stronger electrostatic and van der Waals interactions between trastuzumab and Her2 (the net charge on Her2 is -15, while on trastuzumab and pertuzumab it is +6 and +5 respectively); the buried area at the interface is approximately 1,675 and approximately 1,530 Å^2 ^for trastuzumab and pertuzumab, respectively

- a marked gain in affinity when both Abs are bound simultaneously to the receptor (synergism). This effect arises mainly from the interactions between these two antibodies upon co-localization, with a difference of approximately 27 kcal/mol between isolated and simultaneously bound antibodies (Figure 2C).

**Figure 3:**
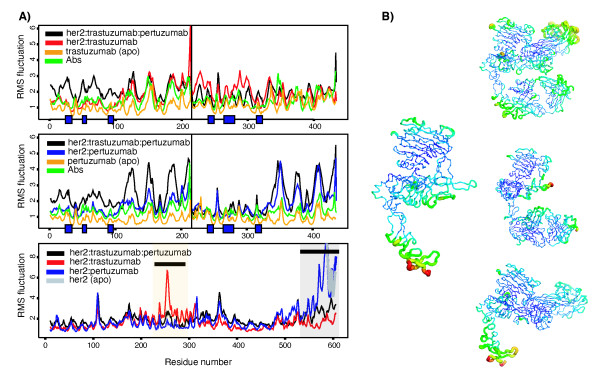
**Structural changes on Her2 ectodomain upon binding of trastuzumab and/or pertuzumab**. To understand the structural changes that occur as a result of the binding of the two antibodies, we analyse the conformational transition/populations using cross 2D-RMSD plots (a plot of RMSD vs RMSD) of the ectodomain of Her2 in the trajectory of the ternary complex of Her2:pertuzumab:trastuzumab and the different apo and binary trajectories. The latter were fitted onto the full receptor structure, except the pertuzumab epitope region, and the rmsd has been calculated for the Cα atoms using the initial structure of the ternary complex simulation as reference. These maps reveal the differences of the various conformations sampled during the simulation with respect to the ternary complex. Different populations of structures are observed in the plots, with structures closest to the conformation adopted for the receptor at the beginning of the ternary simulations shown in blue, to those that are most altered in red (the largest variations are up to approximately 10 Å RMSD). The binding of trastuzumab displaces the conformation of the receptor towards that found in the ternary complex (see plot at the left and density distribution for such a complex). These conformational changes enhance the binding of pertuzumab and thus the affinity of the two antibodies for the receptor is increased, as shown in Table 1, leading to cooperativity.

The computed energetics within these thermodynamic cycles suggest a sequential mechanism, where the initial binding involves trastuzumab (Figure 2A) followed by the binding of pertuzumab (Figure 2B). This seems to be partly driven by the conformational changes triggered upon the binding of trastuzumab that enhance pertuzumab binding (Figure 3) and the new additional contacts formed between trastuzumab and Her2. The enhanced interactions that accrue when the two antibodies are co-localized on Her2 leading to the cooperative effects, may provide a partial explanation for the clinically observed synergy. In order to support the effect of this newly revealed pertuzumab-induced trastuzumab epitope, we have constructed an *in silico *truncated form of Her2, where this β-hairpin has been deleted (T323C-AEDG-T328C), and we find that the binding energetics undergo a marked destabilization (Table 1).

### Abs-induced bending of the Her2 ectodomain

Principal Component Analysis (Additional file 2) combined with dynamical domain motion analysis using DynDom suggests that domains I to III (residues 1 to 480) generally behave as a rigid body, with hinge regions varying in their location in domain IV, depending on the binding partner of the receptor (Additional files 7 and 8). Two different motions are detected in the apo state of Her2; the first one involves a shearing motion between the stalk portion of the receptor and the large rigid-body unit; whereas the second one corresponds to a hinge motion within domain IV. The overall change in conformation involves a large conformational space scanned by the dimerization arm with respect to the membrane surface (with an angle of approximately 50° for the parallel and approximately 78° for the motion perpendicular to the membrane), which represents periscope-like movements that this receptor might execute in search of binding partners. In the case of the ternary complex, the binding of trastuzumab causes a shift in the hinge region along domain IV. This is associated with small swivelling motions across an axis orthogonal to the membrane; if the receptor lies parallel to the membrane [34] then this would represent "out of the membrane" motions. The dimerization arm spans approximately 16 Å in comparison to the apo form which covers approximately 33 Å (measured from the Cα of the atom (Pro252) at the base of this motif). In contrast, in the binary states, the pattern of motion differs: domain IV behaves as a rigid body and only one hinge region is apparent. When trastuzumab is bound, a hinge motion is observed (18°) between the bulky domain I-to-III and domain IV (crystallographic data show approximately 7° in the angle subtended between domains III and IV); the Her2:pertuzumab complex undergoes more complex motions. The deletion in the truncated mutant results in attenuation of two-body rotations along the parallel axis (from 48° in the "full-length" receptor to approximately 28° in the truncated form). In contrast, the bound form of this truncated receptor undergoes a hinged motion spanning approximately 20°.

The overall comparison of the conformations that Her2 ectodomain adopts in the different conditions analysed here indicates that domains I, II and III adopt a relatively fixed interdomain relationship, but their relative orientations with respect to domain IV varies considerably (up to approximately 80°), and appear to be pivoted across the region 504 to 532 that lies between domains III and IV. This is indicative of the plasticity inherent in these receptors and suggests that the extracellular domain can swivel quite considerably across domain IV that itself is constrained by being embedded partly in the membrane.

## Conclusions

In summary, MD simulations suggest that the clinical synergism observed for the two antibodies arises partly from an enhanced affinity that originates in cooperative interactions between these two antibodies when they are co-localized on Her2 and "clamp" it; this may inhibit dimerization and possibly higher oligomerizations with neighbouring receptors. In the presence of trastuzumab, the receptor becomes highly plastic, especially domains I-III, and this appears to promote increased association with pertuzumab. While combining antibodies is a feature that increasingly appears to have major effects in therapy (see, for example, the work of Chao and co-workers [35], which combines two different antibodies against two different cell surface molecules for treatment of non-Hodgkin's lymphoma), a model incorporating the idea that two antibodies bind to the same receptor, albeit at distinct epitopes, is relatively new; indeed there has been only one report of such a feature as highlighted for targeting the IGF-1 receptor [13]. These findings could be tested, for example, in 2D and 3D preclinical cell culture models to explore trastuzumab efficacy [36,37]. While we await experimental investigations of this novel mechanism in Her2, if validated, it offers routes towards the design of new antibodies whereby combinations of trastuzumab and pertuzumab (or parts thereof) may be constructed that have the potential to generate therapeutics with much higher affinities and hopefully efficacies. The technology of generating hybrid antibodies or bi-specific antibodies [38] is known; however, this technique has been used for linking two antibodies that target different molecules. In contrast our hypotheses open the avenue for linking two antibodies that target distinct epitopes on the same molecule.

## Abbreviations

ΔG_bind_: binding free energy; ΔG_ELE_: electrostatics energy; ΔG_NP_: non-polar energy; ΔG_VDW_: van der Waals energy; Abs: antibodies; CDR: complementary determining regions; EGFR: epidermal growth factor receptor; Fab: fragment antigen binding; GB: Generalized-Born; HER2: human epidermal growth factor 2; IGF-1: insulin-like growth factor; LCPO: linear combination of pairwise overlaps; MD: molecular dynamics; MM/GBSA: molecular mechanics/Generalized-Born surface Area; PCA: Principal component analysis; PME: particle mesh Ewald; RTK: receptor tyrosine kinase; SASA: solvent-accessible surface area;

## Competing interests

The authors declare that they have no competing interests.

## Authors' contributions

GF conceived of the study, participated in study design and data interpretation, and drafted the manuscript. MS and JB conceived of the study and revised the manuscript critically for important intellectual content. CV conceived of the study, participated in the analysis and interpretation, and helped to draft the manuscript. All authors read and approved the final manuscript.

## Supplementary Material

Additional file 1 **Supplemental Figure S1**. **Cartoon representation of all the systems involved in the study**; **A) **Apo Her2; **B) **binary complex between Her2:pertuzumab; **C) **binary complex between Her2:trastuzumab; **D) **ternary complex between Her2:pertuzumab:trastuzumab; **E) **pertuzumab; **F) **trastuzumab; **G) **"binary" interaction between trastuzumab:pertuzumab. Trastuzumab has been coloured in black; while pertuzumab is shown in orange. Click here for file

Additional file 2 **Supplemental Figure S2**. **The characterization of the collective protein dynamics have been analyzed using Principal Component Analysis (PCA)**. PCA is a statistical method that highlight similarities and differences in a complex data set by reducing the number of dimensions without losing much of the information. In MD simulations, the application of PCA can separate the configurational space into two subspaces; the essential one containing the functionally important correlated motions (comprising only few degrees of freedom or eigenvectors) and the irrelevant subspace with the independent Gaussian fluctuations with little functional relevance. In this study, PCA has been performed on an ensemble of conformations derived from the different MD simulations. Results of PCA obtained from the diagonalization of the atomic displacement correlation matrix of Cα atoms of the ectodomain from six of the MD simulations carried out in this study (using standard Euclidean distances) are shown here. The resulting Principal Components (PCs) are sorted according to their contribution to the total fluctuation along the ensemble of conformations, and only a small subset of these PCs are necessary to describe the great majority of the total atomic displacements which capture the essential dynamics of the conformational changes occurring in the system (the above so-referred essential subspace). Every figure contains the percentage of the cumulative eigenvalues as a function of the number of eigenvectors considered. The inset includes the projection on the first two eigenvectors of the coordinates extracted from the time interval of 8 to 16 ns. Every figure represents the coordinates for the ectodomain of Her2 from the following simulations: **(A) **apo Her2; **(B) **Her2:pertuzumab:trastuzumab; **(C) **Her2:pertuzumab; **(D) **Her2:trastuzumab; **(E) **apo truncated Her2; **(F) **truncated Her2:pertuzumab:trastuzumab. The application of PCA on data obtained from MD simulations provides a useful way of detecting global, correlated motions of the system that are separate from the tightly-constrained harmonic motions of more constrained atoms. The structures projected on the first eigenvectors have facilitated our understanding of the dynamics of the six systems analysed here (see Supplementary Figure S6). Click here for file

Additional file 3 **Supplemental Figure S3**. **Cα RMS deviation with respect to the starting frame of the 2-ns equilibration phase versus time**. **(A) **apo Her2 simulation; **(B) **ternary complex Her2:trastuzumab:pertuzumab; **(C) **binary complex Her2:trastuzumab; **(D) **binary complex Her2:pertuzumab. The top panel for all the plots represent the RMSD for the whole system; this is broken into the different components in the association in the bottom plots. Click here for file

Additional file 4 **Supplemental Figure S4**. **RSM fluctuation plots and cartoon figures for the different systems of Her2 in the apo-and holo-states with pertuzumab and trastuzumab**. **A) **RMS fluctuations for trastuzumab-containing systems (top); RMS fluctuations for pertuzumab-containing systems (middle panel) and RMS fluctuations for the ectodomain of the receptor in the four different MD simulations; **B) **putty/sausage cartoon representation for apo-receptor (right), Her2 in complex with the two antibodies, with trastuzumab and with pertuzumab (from top to bottom) and colored accordingly to the fluctuation values per residue. The complementarity determining regions (CDR) of both antibodies have been marked in the plot with blue boxes. The trastuzumab and pertuzumab epitopes have been highlighted in shaded boxes in grey and cream and black, respectively. Click here for file

Additional file 5 **Supplemental Table S1**. **Cα-Cα distances for the residues at the receptor:Ab interfaces detected along the simulations between Her2 and the two Abs: pertuzumab and trastuzumab**. The different shading boxes indicate the CDR of the antibodies. Click here for file

Additional file 6 **Supplemental Figure S5**. **Interactions between the two antibodies and new epitope revealed in the MD simulations**. **(A) **Density distributions for the intermolecular Cα-Cα distance between Glu294 and Asp295 (labeled in the Figure C) in the pertuzumab-induced trastuzumab epitope of the receptor and their putative interacting residues at the interface in trastuzumab. The dashed lines represent the population in the Her2:pertuzumab:trastuzumab simulation, while the straight lines show the distribution found in the Her2:trastuzumab simulation. **(B) **Density distribution for the intermolecular Cα-Cα distance between residues in the different Abs. In horizontal lines the average distance for the ternary (red) and binary (blue) simulations are shown. **(C) **Schematic representation of the residues for which the distances have been measured. The color coding is the same as in Figure S1. Click here for file

Additional file 7 **Supplemental Figure S6**. **Cartoon representation of the maximum and minimum conformations of Her2 along the first eigenvector extracted from the PCA analysis and colored according to the Dyndom output **(see Additional file 8) for the following simulations: **(A) **apo Her2; **(B) **Her2:pertuzumab:trastuzumab; **(C) **Her2:pertuzumab; **(D) **Her2:trastuzumab; **(E) **apo truncated Her2; **(F) **truncated Her2:pertuzumab:trastuzumab. All the models were fit onto the last 20 residues of the ectodomain. Click here for file

Additional file 8 **Supplemental Table S2**. **Dynamical domains and hinge bending residues **determined by the program DYNDOM for the superposition of the Her2 receptor of the trajectories of this domain in the apo, dimeric and trimeric forms with trastuzumab and pertuzumab. Click here for file
