# Supplementary figures and images for: Synergy between trastuzumab and pertuzumab for human epidermal growth factor 2 (Her2) from colocalization: an in silico based mechanism
Source: Breast Cancer Res. 2011 May 22;13(3):R54. doi: 10.1186/bcr2888 (PMC3218942; doi:10.1186/bcr2888)

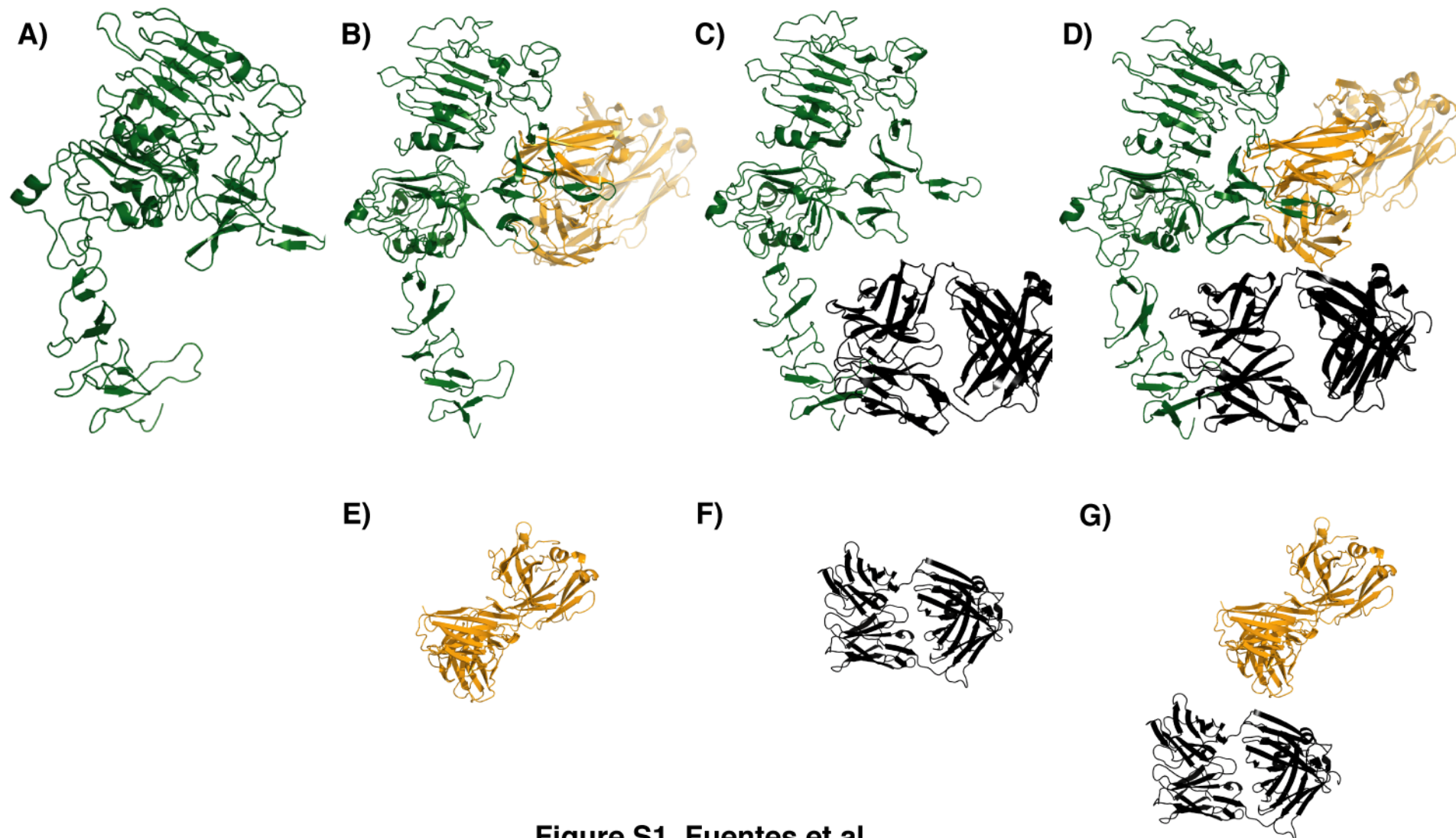

**Figure S1. Fuentes et al.**

Supplement: Additional file 1 — Supplemental Figure S1. Cartoon representation of all the systems involved in the study; A) Apo Her2; B) binary complex between Her2:pertuzumab; C) binary complex between Her2:trastuzumab; D) ternary complex between Her2:pertuzumab:trastuzumab; E) pertuzumab; F) trastuzumab; G) "binary" interaction between trastuzumab:pertuzumab. Trastuzumab has been coloured in black; while pertuzumab is shown in orange. [file bcr2888-S1.PDF]

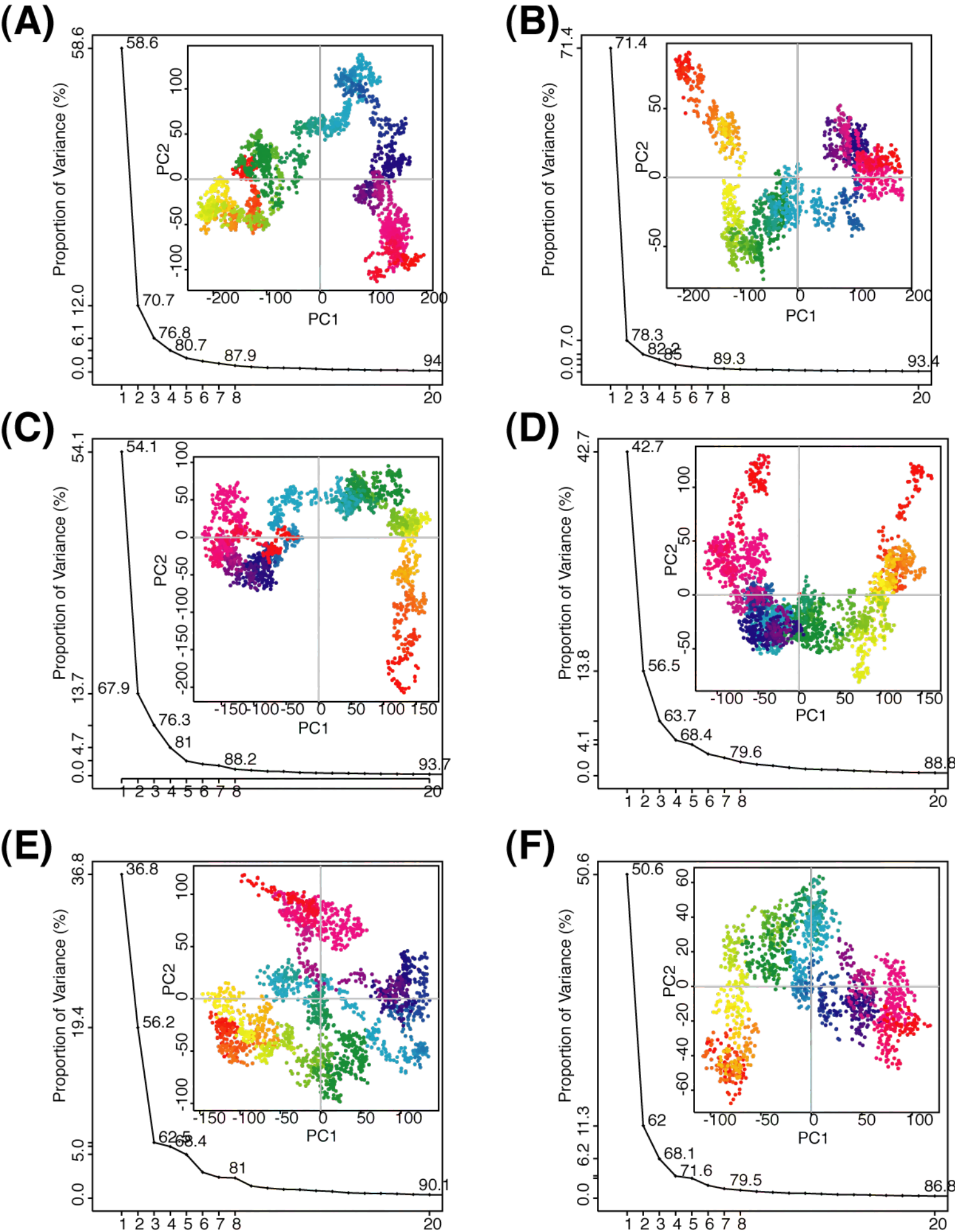

**Figure S5. Fuentes et al.**

Supplement: Additional file 2 — Supplemental Figure S2. The characterization of the collective protein dynamics have been analyzed using Principal Component Analysis (PCA). PCA is a statistical method that highlight similarities and differences in a complex data set by reducing the number of dimensions without losing much of the information. In MD simulations, the application of PCA can separate the configurational space into two subspaces; the essential one containing the functionally important correlated motions (comprising only few degrees of freedom or eigenvectors) and the irrelevant subspace with the independent Gaussian fluctuations with little functional relevance. In this study, PCA has been performed on an ensemble of conformations derived from the different MD simulations. Results of PCA obtained from the diagonalization of the atomic displacement correlation matrix of Cα atoms of the ectodomain from six of the MD simulations carried out in this study (using standard Euclidean distances) are shown here. The resulting Principal Components (PCs) are sorted according to their contribution to the total fluctuation along the ensemble of conformations, and only a small subset of these PCs are necessary to describe the great majority of the total atomic displacements which capture the essential dynamics of the conformational changes occurring in the system (the above so-referred essential subspace). Every figure contains the percentage of the cumulative eigenvalues as a function of the number of eigenvectors considered. The inset includes the projection on the first two eigenvectors of the coordinates extracted from the time interval of 8 to 16 ns. Every figure represents the coordinates for the ectodomain of Her2 from the following simulations: (A) apo Her2; (B) Her2:pertuzumab:trastuzumab; (C) Her2:pertuzumab; (D) Her2:trastuzumab; (E) apo truncated Her2; (F) truncated Her2:pertuzumab:trastuzumab. The application of PCA on data obtained from MD simulations provides a useful way of detecting [file bcr2888-S2.PDF]

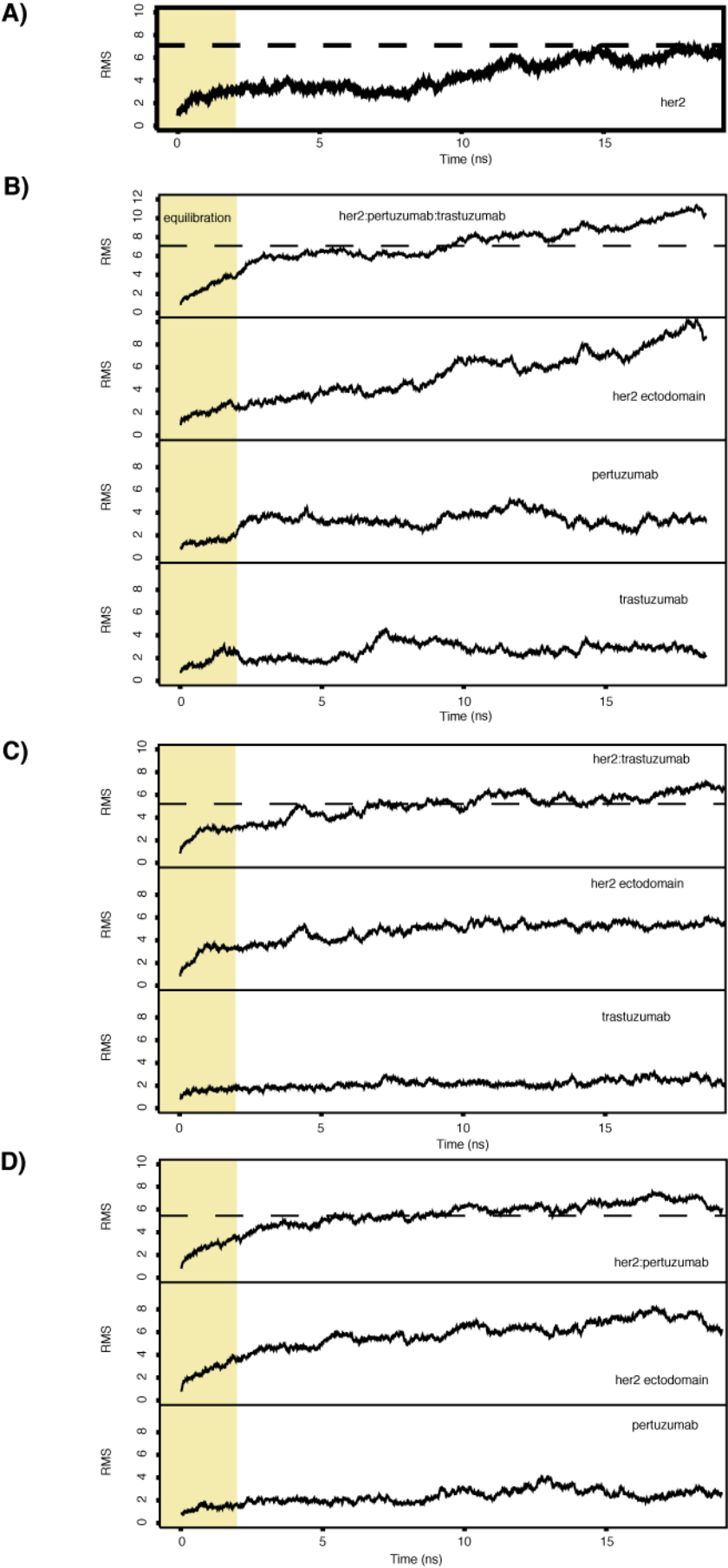

**Figure S2. Fuentes et al.**

Supplement: Additional file 3 — Supplemental Figure S3. Cα RMS deviation with respect to the starting frame of the 2-ns equilibration phase versus time. (A) apo Her2 simulation; (B) ternary complex Her2:trastuzumab:pertuzumab; (C) binary complex Her2:trastuzumab; (D) binary complex Her2:pertuzumab. The top panel for all the plots represent the RMSD for the whole system; this is broken into the different components in the association in the bottom plots. [file bcr2888-S3.PDF]

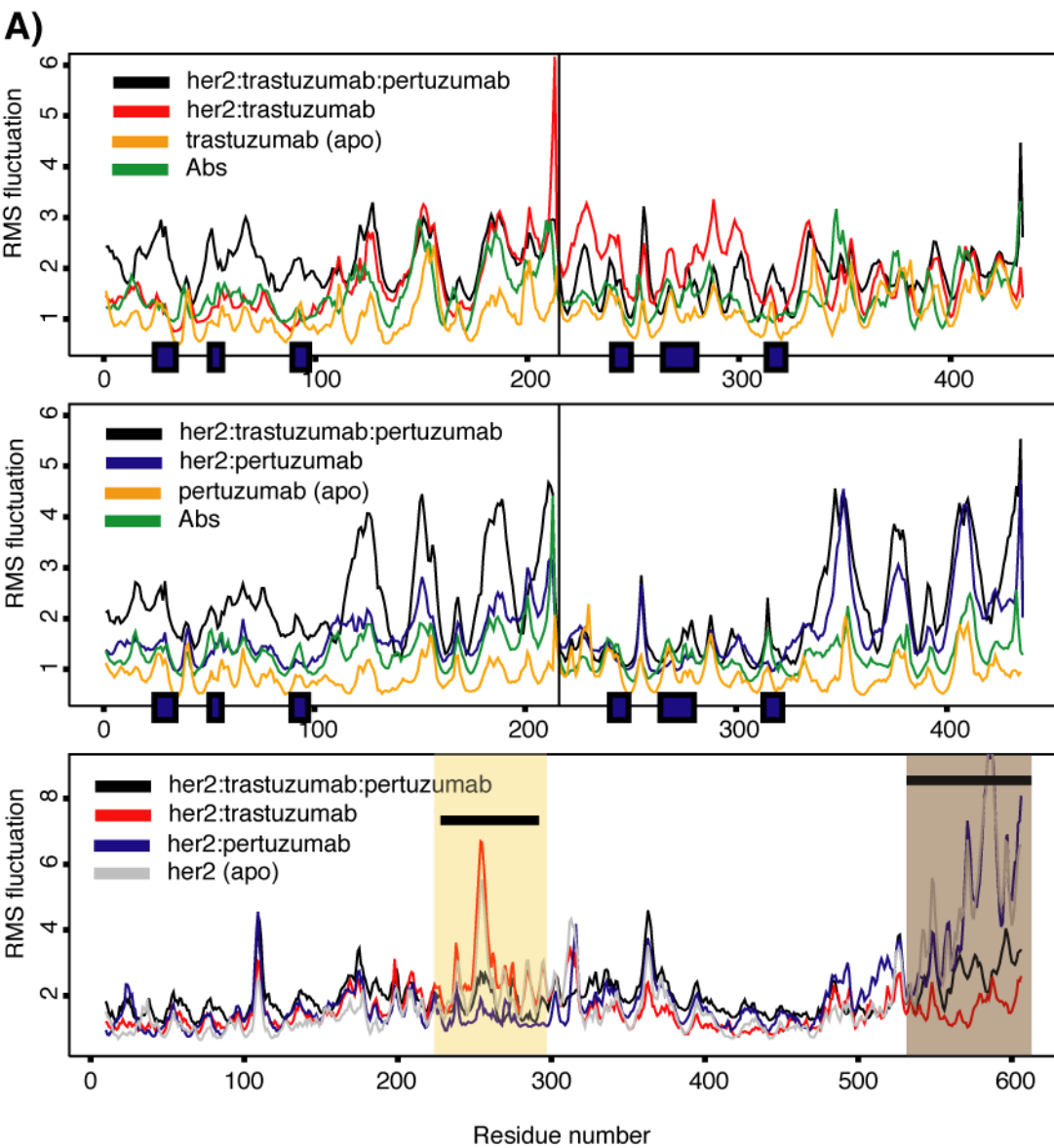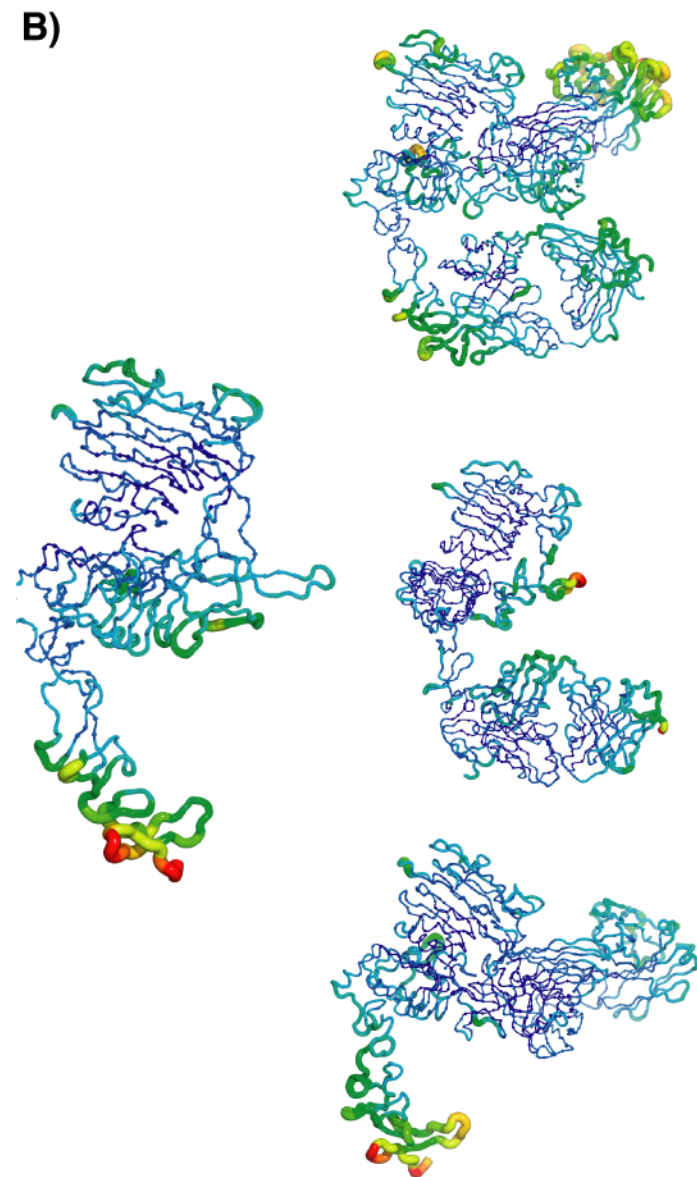

**Figure S3. Fuentes et al.**

Supplement: Additional file 4 — Supplemental Figure S4. RSM fluctuation plots and cartoon figures for the different systems of Her2 in the apo-and holo-states with pertuzumab and trastuzumab. A) RMS fluctuations for trastuzumab-containing systems (top); RMS fluctuations for pertuzumab-containing systems (middle panel) and RMS fluctuations for the ectodomain of the receptor in the four different MD simulations; B) putty/sausage cartoon representation for apo-receptor (right), Her2 in complex with the two antibodies, with trastuzumab and with pertuzumab (from top to bottom) and colored accordingly to the fluctuation values per residue. The complementarity determining regions (CDR) of both antibodies have been marked in the plot with blue boxes. The trastuzumab and pertuzumab epitopes have been highlighted in shaded boxes in grey and cream and black, respectively. [file bcr2888-S4.PDF]

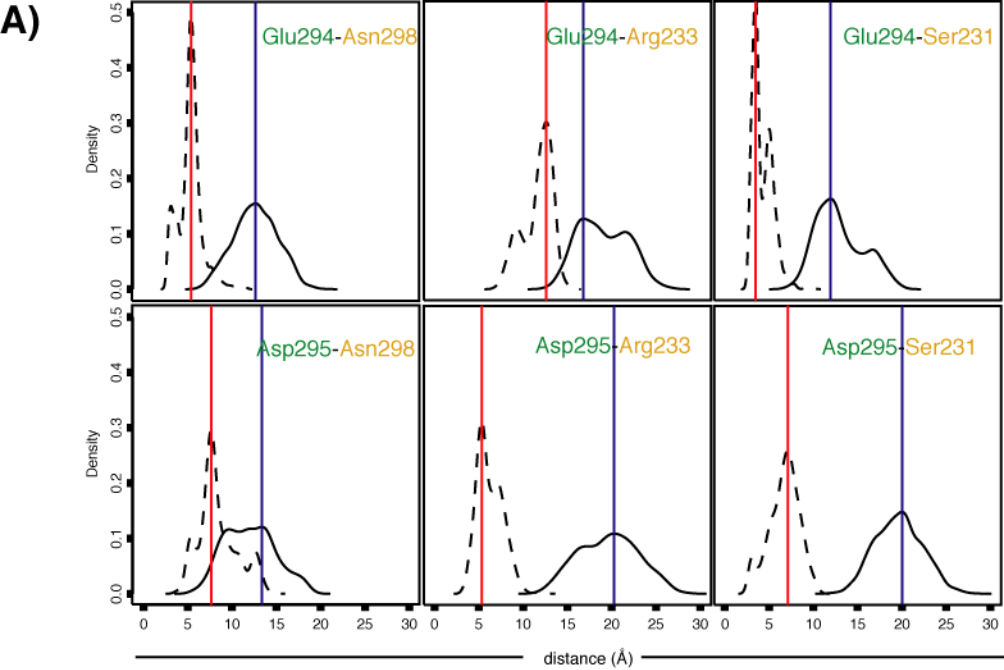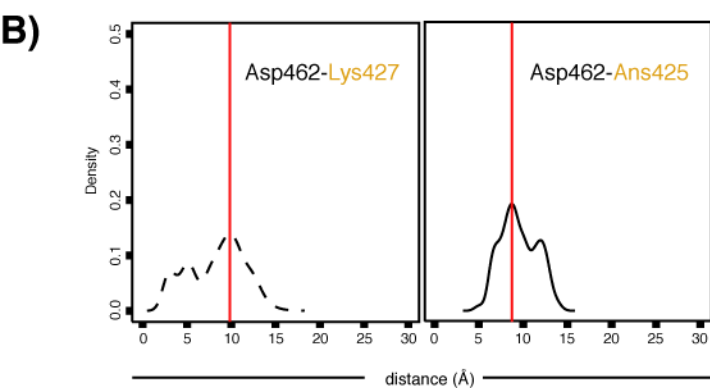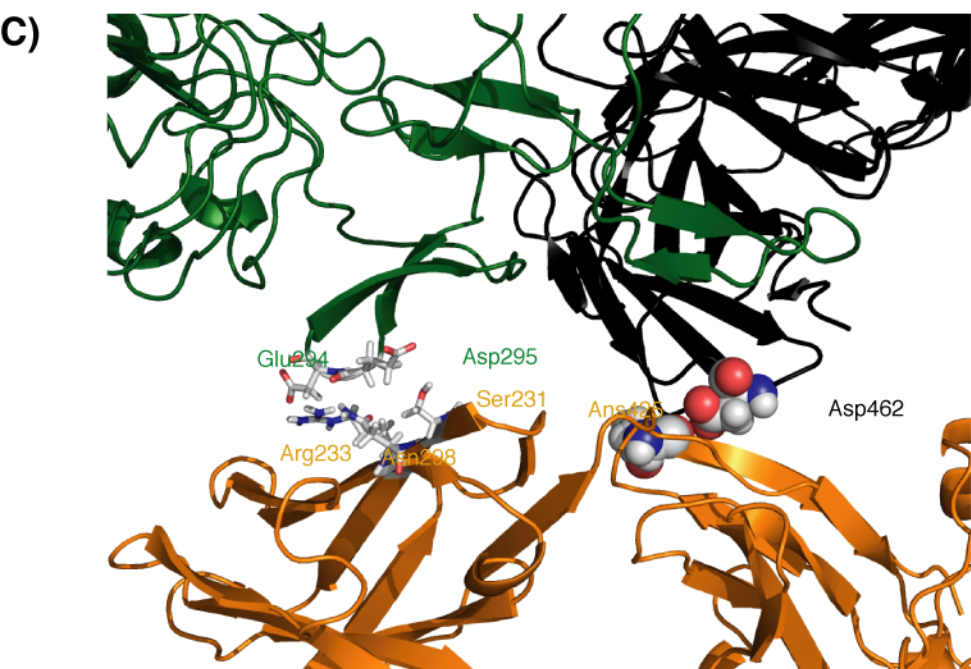

**Figure S4. Fuentes et al.**

Supplement: Additional file 6 — Supplemental Figure S5. Interactions between the two antibodies and new epitope revealed in the MD simulations. (A) Density distributions for the intermolecular Cα-Cα distance between Glu294 and Asp295 (labeled in the Figure C) in the pertuzumab-induced trastuzumab epitope of the receptor and their putative interacting residues at the interface in trastuzumab. The dashed lines represent the population in the Her2:pertuzumab:trastuzumab simulation, while the straight lines show the distribution found in the Her2:trastuzumab simulation. (B) Density distribution for the intermolecular Cα-Cα distance between residues in the different Abs. In horizontal lines the average distance for the ternary (red) and binary (blue) simulations are shown. (C) Schematic representation of the residues for which the distances have been measured. The color coding is the same as in Figure S1. [file bcr2888-S6.PDF]

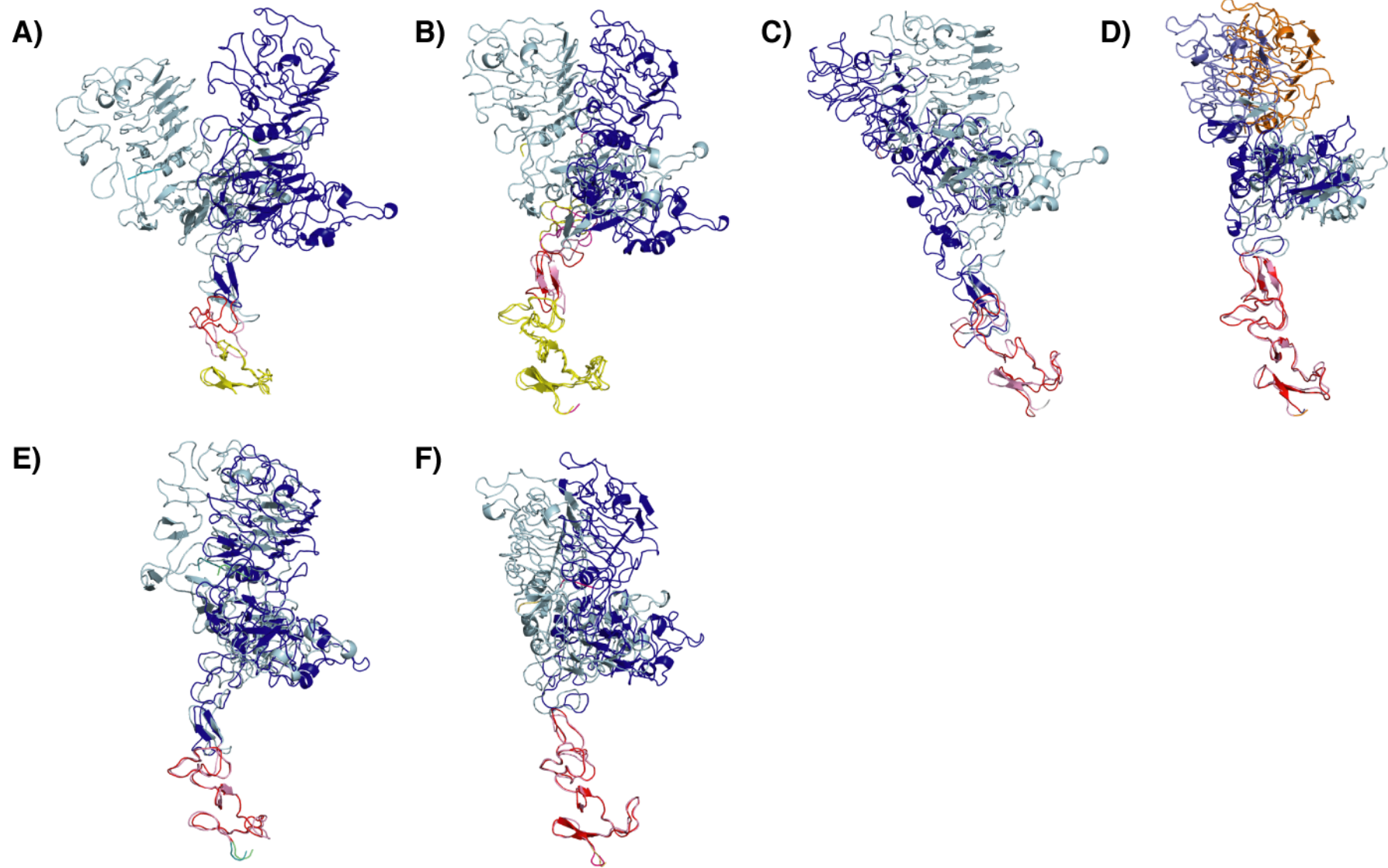

**Figure S6. Fuentes et al.**

Supplement: Additional file 7 — Supplemental Figure S6. Cartoon representation of the maximum and minimum conformations of Her2 along the first eigenvector extracted from the PCA analysis and colored according to the Dyndom output (see Additional file 8) for the following simulations: (A) apo Her2; (B) Her2:pertuzumab:trastuzumab; (C) Her2:pertuzumab; (D) Her2:trastuzumab; (E) apo truncated Her2; (F) truncated Her2:pertuzumab:trastuzumab. All the models were fit onto the last 20 residues of the ectodomain. [file bcr2888-S7.PDF]
