# Supplementary material for: Synergy between trastuzumab and pertuzumab for human epidermal growth factor 2 (Her2) from colocalization: an in silico based mechanism
Source: Breast Cancer Res. 2011 May 22;13(3):R54. doi: 10.1186/bcr2888 (PMC3218942; doi:10.1186/bcr2888)
Supplement: Additional file 5 — Supplemental Table S1. Cα-Cα distances for the residues at the receptor:Ab interfaces detected along the simulations between Her2 and the two Abs: pertuzumab and trastuzumab. The different shading boxes indicate the CDR of the antibodies. [file bcr2888-S5.DOC]

Table S1

| Her2 | Abs | Her2-P(-T) | Her2-P | Her2-(P)-T | Her2-T | trunc-her2-P-T |
| --- | --- | --- | --- | --- | --- | --- |
| HIS287 | THR490 |  | 37.89 |  |  |  |
| SER300 | SER484 |  |  |  |  | 62.70 |
| SER304 | SER484 | 35.18 | 35.80 |  |  |  |
| SER300 | TYR487 |  |  |  |  | 34.48 |
| LYS305 | TYR487 | 34.27 | 36.58 |  |  |  |
| HIS287 | THR490 |  | 37.89 |  |  |  |
| THR247 | TYR528 | 30.61 | 30.82 |  |  | 33.94 |
| SER279 | THR678 | 54.90 | 72.80 |  |  | 96.64 |
| SER279 | ASP679 | 88.92 | 81.45 |  |  | 99.87 |
| PRO285 | ASP679 |  | 73.43 |  |  |  |
| THR275 | ASP679 | 39.07 |  |  |  | 72.78 |
| VAL277 | ASP679 | 93.97 | 99.06 |  |  | 92.41 |
| VAL277 | TYR680 | 88.25 | 95.86 |  |  | 89.58 |
| VAL277 | THR681 | 35.48 | 39.78 |  |  | 31.99 |
| VAL277 | ASN700 | 80.71 | 51.57 |  |  | 87.77 |
| HIS236 | ASN702 | 99.70 | 99.74 |  |  | 100 |
| CYS237 | ASN702 | 99.76 | 99.74 |  |  | 100 |
| THR259 | ASN702 |  | 30.19 |  |  | 33.40 |
| VAL277 | ASN702 | 45.47 |  |  |  |  |
| GLY278 | ASN702 | 77.60 | 95.96 |  |  | 97.51 |
| HIS236 | SER703 | 100.00 | 100.00 |  |  | 100 |
| CYS237 | SER703 | 100.00 | 100.00 |  |  | 100 |
| PRO238 | SER703 | 99.94 | 99.95 |  |  | 99.87 |
| ALA239 | SER703 | 100.00 | 99.74 |  |  | 100 |
| HIS236 | GLY704 | 93.12 | 97.06 |  |  | 97.45 |
| PHE248 | ILE707 | 39.99 |  |  |  | 57.06 |
| HIS226 | SER723 | 43.40 |  |  |  |  |
| LEU286 | LEU748 | 39.14 |  |  |  |  |
| PRO285 | GLY749 | 51.43 | 30.40 |  |  |  |
| LEU286 | GLY749 | 31.35 |  |  |  |  |
| LYS302 | GLY749 |  | 37.68 |  |  |  |
| ASP276 | PRO750 | 57.64 | 77.78 |  |  |  |
| VAL277 | PRO750 |  | 38.57 |  |  |  |
| LYS302 | PRO750 | 61.05 |  |  |  |  |
| VAL277 | GLN759 |  |  |  |  | 55.44 |
| SER562 | THR31 |  |  | 61.90 | 75.27 | 100 |
| GLN593 | THR31 |  |  |  | 63.24 |  |
| SER562 | ALA32 |  |  | 72.85 | 100.00 | 100 |
| SER563 | ALA32 |  |  | 48.02 | 83.89 | 68.88 |
| SER562 | SER50 |  |  |  |  | 74.40 |
| GLN593 | SER50 |  |  |  | 51.85 |  |
| PRO594 | SER50 |  |  |  | 35.19 |  |
| LEU577 | SER56 |  |  | 75.23 | 59.63 |  |
| SER578 | SER56 |  |  | 97.75 | 72.59 | 98.59 |
| TYR579 | SER56 |  |  |  |  | 93.41 |
| MET580 | SER56 |  |  |  |  | 96.10 |
| SER563 | HID91 |  |  | 35.85 | 95.45 | 97.78 |
| SER562 | TYR92 |  |  | 71.15 | 97.37 | 100 |
| SER563 | TYR92 |  |  | 88.86 | 100.00 | 100 |
| ASP551 | THR93 |  |  | 49.00 | 99.53 |  |
| SER563 | THR93 |  |  | 62.08 | 98.17 | 97.51 |
| GLN552 | THR93 |  |  |  | 96.64 |  |
| ASP551 | THR94 |  |  |  | 96.22 |  |
| GLN552 | THR94 |  |  |  | 95.92 |  |
| GLU294 | SER231 |  |  | 64.46 |  |  |
| GLU294 | ASN298 |  |  | 44.92 |  |  |
| PRO575 | GLY314 |  |  | 99.33 | 76.41 | 99.60 |
| ASP576 | GLY314 |  |  | 49.06 | 56.69 | 98.86 |
| LEU577 | GLY314 |  |  |  |  | 33.67 |
| PRO575 | GLY315 |  |  | 87.64 | 55.76 | 100 |
| ASP576 | GLY315 |  |  | 34.39 | 34.30 | 98.05 |
| ASP561 | GLY317 |  |  |  | 31.45 |  |
| PRO570 | GLY317 |  |  |  |  | 49.33 |
| VAL1443 | GLY317 |  |  | 69.20 | 71.65 |  |
| PRO575 | PHE318 |  |  |  | 37.65 | 42.20 |
| SER563 | TYR319 |  |  |  | 44.71 | 47.65 |

Abs: antibodies (here specifically refers to pertuzumab and trastuzumab); Her2-P(-T): Her2-pertuzumab complex in the conformation where trastuzumab is also bound; Her2-P: Her2-pertuzumab complex in the conformation where only this antibody is bound; Her2-(P)-T: Her2-trastuzumab complex in the conformation where pertuzumab is also colocalized on the receptor; Her2-T: Her2-trastuzumab complex in the conformation where only trastuzumab is bound to receptor; trunc-Her2-P-T: truncated form of Her2 bound to both antibodies.
