# Supplementary material for: Synergy between trastuzumab and pertuzumab for human epidermal growth factor 2 (Her2) from colocalization: an in silico based mechanism
Source: Breast Cancer Res. 2011 May 22;13(3):R54. doi: 10.1186/bcr2888 (PMC3218942; doi:10.1186/bcr2888)
Supplement: Additional file 8 — Supplemental Table S2. Dynamical domains and hinge bending residues determined by the program DYNDOM for the superposition of the Her2 receptor of the trajectories of this domain in the apo, dimeric and trimeric forms with trastuzumab and pertuzumab. [file bcr2888-S8.DOC]

Table S2

| System | Domain 1 | Domain 2 | bending residues | rotation (º) | Translation along axis (Å) | % closure motion (º) |
| --- | --- | --- | --- | --- | --- | --- |
| Her2 (apo) | 5 - 531 | 532 - 556 | 504 - 532 | 48.4 | 2.8 | 21.0 |
|  | 532-556 | 557 - 594 | 556 - 558 | 77.6 | -2.0 | 66.0 |
| Her2-P-T | 3-447 | 503-531 | 447-503 | 18.2 | 1.6 | 20.2 |
|  | 503-531 | 532-596 | 531-532 | 27.5 | 0.0 | 2.4 |
| Her2-T | 227-509 | 510-596 | 509-510 | 18.7 | -0.7 | 90.3 |
| Her2-P | 3-539 | 540-596 | 529-540 | 36.4 | 1.3 | 53.6 |
| trunc-Her2 | 5 - 524 | 525-587 | 505-528 | 28.1 | -0.4 | 9.4 |
| trunc-Her2-Abs | 5-504 | 505-587 | 503-506 | 20.5 | 0.0 | 94.8 |

Her2-P-T: Her2 bound to pertuzumab and trastuzumab; Her2-T: Her2 in complex with trastuzumab; Her2-P: Her2 bound to pertuzumab; trunc-Her2: truncated form of Her2; trunc-Her2-Abs: truncated form of Her2 bound to both antibodies, trastuzumab and pertuzumab.
